# SARS-CoV-2: Outline, Prevention, and Decontamination

**DOI:** 10.3390/pathogens10020114

**Published:** 2021-01-23

**Authors:** Edyta Janik, Maciej Bartos, Marcin Niemcewicz, Leslaw Gorniak, Michal Bijak

**Affiliations:** 1 Biohazard Prevention Centre, Faculty of Biology and Environmental Protection, University of Lodz, Pomorska 141/143, 90-236 Lodz, Poland. edyta.janik@unilodz.eu (E.J.); marcin.niemcewicz@biol.uni.lodz.pl (M.N.); leslaw.gorniak@biol.uni.lodz.pl (L.G.); 2 Department of Biodiversity Studies and Bioeducation, Faculty of Biology and Environmental Protection, University of Lodz, Banacha 1/3, 90-237 Lodz, Poland. maciej.bartos@biol.uni.lodz.pl

**Keywords:** COVID-19, SARS-CoV-2, personal protection, decontamination

## Abstract

The new coronavirus began to spread around the world in late 2019. Initially, it was found only in China, but in the following days there were reported cases of infections in other countries. Subsequently, based on taxonomy, phylogeny, and accepted practice, the virus was officially designated as severe acute respiratory syndrome coronavirus 2 (SARS-CoV-2). As a result of the rapid spread of SARS-CoV-2 in different countries around the world, on March 11, 2020, the World Health Organization (WHO) announced a status change in the disease caused by this coronavirus—from an epidemic to a pandemic disease. Although the world is taking unprecedented efforts to control the spread of SARS-CoV-2, the number of confirmed cases is rising. Therefore, effective preventive measures are needed in order to limit the spread of illness. The prevention measures are mainly based on information on the virus transmission routes, its environmental stability, and persistence on commonly touched surfaces. Social distancing, mask usage, and good hygiene practice are the most important recommendations for general public. Healthcare professionals who are directly involved in SARS-CoV-2 patients care are more exposed to virus infection and additional protection measures are necessary, including protective suits, aprons, face shields, goggles, and gloves. Due to the stability of SARS-CoV-2 on different surfaces, such as glass, paper, or wood, proper disinfection is crucial. Several studies have shown that despite the virus’s stability, it is sensitive to various disinfectants, such as ethanol, isopropanol, sodium hypochlorite, or hydrogen peroxide. These findings underline the importance of having comprehensive knowledge about SARS-CoV-2 and multidirectional strategies in order to limit the spread of the virus. This review is a summary of the most important information about SARS-CoV-2, such as its stability on different surfaces, protection strategies, and decontamination options.

## 1. Introduction

In late autumn 2019 in Wuhan, Hubei Province, China, local health services reported cases of patients with pneumonia of unknown origin. Symptoms of the first patient included malaise, fever, dyspnea, and dry cough, and were diagnosed as viral pneumonia. A whole genome sequence of a sample taken from the patient showed that the causative agent of the pneumonia is a novel coronavirus, making it the seventh member of the coronavirus family able to infect humans [1,2]. Furthermore, the International Committee on Taxonomy of Viruses (ICTV) has officially named the virus as severe acute respiratory syndrome coronavirus 2—SARS-CoV-2 [3]. Following the virus spreading, the World Health Organization (WHO) officially announced the SARS-CoV-2 pandemic on 11 March 2020 [4], and officially named the disease caused by SARS-CoV-2 as Coronavirus Disease 2019 (COVID-19). Since December 2019, the virus has spread to over 200 countries and territories. According to the WHO report, at 10 January 2021, over 87.5 million confirmed cases of COVID-19, including more than 1,900,000 deaths, were observed [5]. These data indicate that COVID-19 is highly contagious and global measures have been applied in order to limit the spread of the virus [6]. At the beginning, the WHO’s general recommendations to the general public was to wear a mask in public places and apply hygiene practices as the available protection options [7,8]. Simultaneously, various medical therapies have been included in the COVID-19 Treatment Guidelines published by the National Institutes of Health (NIH). Antiviral agents, including remdesivir, chloroquine, hydroxychloroquine, and lopinavir/ritonavir, were recommended in antiviral therapy [9]. In October 2020, the U.S. Food and Drug Administration (FDA) also approved remdesivir as a first treatment option for COVID-19 [10]. Remdesivir is an inhibitor of the viral RNA-dependent RNA polymerase enzyme and was identified as a possible therapeutic candidate for COVID-19 due to its ability to inhibit SARS-CoV-2 in vitro [11]. Beigel et al. have showed that remdesivir was superior to the placebo in reducing recovery time in adults. The results also indicate that treatment with remdesivir may prevent disease progression to a more severe respiratory distress [12]. In November 2020, the FDA authorized baricitinib in combination with remdesivir as another treatment option [13]. Baricitinib is a selective inhibitor of Janus kinase (JAK) 1 and 2, which blocks the activity of one or more of this specific family of enzymes that interferes with the pathway that leads to inflammation [14]. Kalil et al. have showed that the combination of baricitinib and remdesivir was superior to remdesivir alone in reducing recovery time and accelerating clinical status improvement [15]. Immune-based therapy is a different approach in COVID-19 treatment. Different blood-derived products and immunomodulators used in COVID-19 treatment were discussed. Systemic transfusion of convalescent plasma (CP) collected from healthy donors who recovered from SARS CoV-2 seems to be an effective option [16]. A study conducted on patients with severe COVID-19 has shown that in all CP-transfused patients who obtained SARS-CoV-2 serum, an increase in oxygen saturation and lymphocyte counts and an improvement in their liver function and C-reactive protein (CRP) were observed [17,18]. It has been shown that monoclonal antibody therapy can also be effective in COVID-19 treatment [19,20,21]. Furthermore, also in November 2020, the FDA issued an emergency use authorization (EUA) for the investigational monoclonal antibody therapy containing bamlanivimab, casirivimab, and imdevimab for administration in specific COVID-19 cases. Bamlanivimab is specifically directed against the spike (S) protein of SARS-CoV-2 and designed to block the virus’ attachment and entry into the cells. Casirivimab and imdevimab have to be administered together in mild and moderate cases. Their mechanism of action is the same as that of bamlanivimab [22]. Concurrently, tremendous efforts and financial support were put into vaccine elaboration, as an efficacious vaccine is considered essential in curbing the spread of this virus, which would prevent further morbidity and health systems collapsing. At present, two mRNA vaccines are authorized and recommended to prevent COVID-19: Pfizer–BioNTech (BNT162b2) and Moderna COVID-19 (mRNA-1273) vaccines [23,24]. The BNT162b2 is a lipid nanoparticle–formulated, nucleoside-modified RNA (modRNA) vaccine encoding the SARS-CoV-2 full-length spike, modified by two proline mutations to lock it in the prefusion conformation [25]. Findings from clinical trials indicate that the Pfizer–BioNTech COVID-19 vaccine was 95.0% effective in preventing symptomatic laboratory-confirmed COVID-19 in people without evidence of previous SARS-CoV-2 infection. Vaccination with the Pfizer–BioNTech COVID-19 vaccine requires a 2-dose series administered intramuscularly, 3 weeks apart [26]. The mRNA-1273 is a lipid-nanoparticle (LNP)-encapsulated mRNA vaccine expressing the prefusion-stabilized spike glycoprotein. In clinical trials, the mRNA-1273 vaccine showed 94.1% efficacy at preventing COVID-19 disease, including severe disease cases [27]. Vaccination with the Moderna COVID-19 vaccine also requires two injections administered intramuscularly; however, it is recommended that the booster dose should be injected after 4 weeks from the first one [28]. The main difference between these two are storage and transport. The BNT162b2 requires −70 °C, while mRNA-1273 can be transported at −20 °C. In a regular refrigerator, the Pfizer–BioNTech vaccine can placed for up to five days, while the Moderna one can stay up to 30 days. The following are differences: BNT162b2 can be administered to people over 16 years old, while mRNA-1273 is suitable for people 18 years and older.

In conclusion, the world is facing one of the biggest health threats. Many institutes, hospitals, and scientists are involved in order to develop a strategy to control the spread of SARS-CoV-2 and effectively treat COVID-19. Thus, the purpose of this review is to describe the SARS-CoV-2 structure, mechanism of action, transmission routes, characteristics of the virus’ stability, and protection and decontamination options.

## 2. Historical Overview of Pandemics

Epidemics and pandemics have accompanied humanity since ancient times. They usually appeared suddenly and spread at a rapid pace. The most tragic of these were fatal to millions of people around the world [29,30]. Among the many well known and most tragic in consequences is The Plague of Athens, which occurred in 430–426 BC through the Peloponnesian War. The cause of the plague remains under continuous debate. Most often cited as the cause are influenza, typhoid fever, epidemic typhus, smallpox, bubonic plague, and measles. The total number of deaths has been estimated as 5 million [31,32]. The next pandemic was the Justinianic Plague. It was caused by *Yersinia pestis*, which was initiated in the mid-sixth century AD. This pandemic is the earliest well documented example of a plague outbreak [33,34]. Based on historical and molecular studies, it is estimated that the Justinianic Plague death rate was as high as 100 million [35]. The following pandemic in human history was a pandemic of bubonic plague in the 14th century—the Black Death. During fifty years of its reign, the pandemic killed as many as 150 million human beings [36,37]. The subsequent pandemic, Spanish Flu, appeared in 1918 and was the first pandemic that occurred at a time when medicines were already developed and the nature and course of diseases were studied. The pandemic was caused by one of the influenza virus strains—H1N1. It is estimated that a third of the world’s population was affected by this virus [33,38]. The number of deaths was enormous—over 50 million, probably reaching close to 100 million deceased. It is estimated that during one year this virus has caused the death of more people than the Black Death did within a century [39]. Other serious pandemics of the 20th century were the Asian flu and the Hong Kong flu. The Asian flu broke out in 1957 and caused about one to two million deaths worldwide. This virus was found to be a subtype of the H2N2 influenza A virus, as an effect of the combination of the human and avian influenza viruses. The Hong Kong flu was caused by subtype H3N2, resulting from notable genetic reassortment that allowed to infect birds and mammals; it was the primary reason for over one million confirmed deaths worldwide [40,41,42]. During the reign of various pandemics, many attempts were undertaken in order to improve the treatment and prevent the outbreak of diseases. Medicines were not as developed as they are now and different treatment approaches were tested. It is known from historical sources that air purification with juniper, diet, and sleep were strongly recommended during The Black Death pandemic. Another way to prevent the disease spreading was to isolate infected people and quarantine them. Herbs, vinegar, and essential oils were used as medicines. Medics wore characteristic outfits with face masks filled with various herbs in order to protect themselves against infection and stench. These actions resulted from, among others, insufficient knowledge concerning bacteria and viruses and low hygiene standards [43]. Now, disease treatment is easier than it was 100 years ago [44]. Despite the significant development of medicine and pharmacy, the treatment of certain diseases, especially those that develop suddenly and spread rapidly, is very challenging.

## 3. Coronavirus Family

Coronaviruses (CoVs) are enveloped, positive-sense, single-stranded RNA viruses. The coronavirus genome size ranges from approximately 26 to 32 kilobases, which is the largest known genome of an RNA virus. CoVs are classified as alphacoronaviruses and betacoronaviruses, both of which originate from bats and occur mainly in mammals, such as bats, rodents, civets, and humans; and gammacoronaviruses and deltacoronaviruses, both of which have their gene source from avian genes and are mainly present in birds [45,46,47]. In the structure of the CoVs is present non-segmented genomes, which share a similar organization. About two thirds of the genome contains two large overlapping open reading frames (ORF1a and ORF1b) coding replicase polyproteins—pp1a and pp1ab. Those polyproteins are then processed to produce 16 nonstructural proteins, defined nsp1-16. The structural genes encode various structural proteins [46,48]. They cause respiratory, hepatic, enteric, and neurologic diseases. By the end of 2019, six species of coronaviruses were identified that are able to cause diseases in humans. Four of them (229E, OC43, HKU1, and NL63) are common and usually cause typical symptoms of a cold-related illness in humans with normal immunity. The other two strains—Middle East respiratory syndrome coronavirus (MERS-CoV) and severe acute respiratory syndrome coronavirus (SARS-CoV)—are zoonotic in origin and are associated with fatal diseases [49,50,51]. SARS-CoV caused outbreaks of severe acute respiratory syndrome in 2002 and 2003 in Guangdong Province, southern China, which spread to South-East Asia North America, and Europe, starting the first 21st century pandemic [52]. MERS-CoV appeared in Saudi Arabia in 2012 and led to an epidemic in the Middle East. The virus produced severe and progressive pneumonia, often accompanied by renal failure [53,54]. An analysis has also indicated the similarity of SARS-CoV-2 to SARS-CoV, at ~79%, and to MERS-CoV, at ~50% [55,56]. In comparison to SARS-CoV and MERS-CoV, which appeared in 2002 and 2012, respectively, SARS-CoV-2 has rapidly spread around the world. The estimated fatality rate in confirmed cases reached 6.6% for SARS-CoV-2, which is lower than the fatality rate for SARS-CoV and MERS-CoV, which was 9.6% and 34.3%, respectively [57,58]. 

## 4. SARS-CoV-2—Structure and Transmission Routes

**Figure 1:**
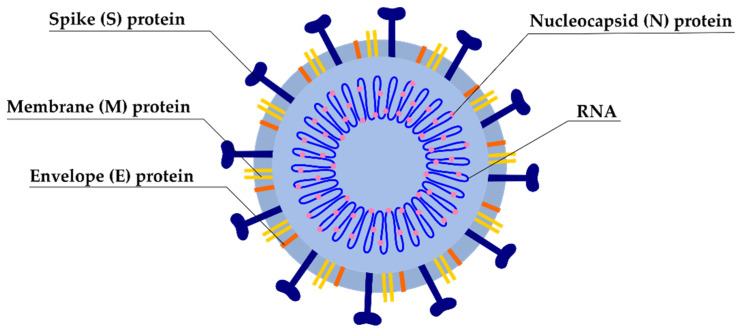
Schematic structure of SARS-CoV-2.

SARS-CoV-2 particles are enveloped with prominent spikes. The virions are spherical and approximately range in size from 65 to 125 nm [59]. SARS-CoV-2 has four major structural proteins, including spike glycoprotein (S), small envelope (E), membrane glycoprotein (M), and nucleocapsid (N) protein, as well as 16 nonstructural proteins (NSPs) and several accessory proteins (Figure 1) [60]. Among them, the S glycoprotein is a major protein, which plays a crucial role in viral attachment, fusion, entry, and transmission [61]. S protein is cleaved in the host cell by the furin-like protease into two subunits—called S1 and S2. The first, S1, is responsible for the determination of the host virus range and cellular tropism with the receptor-binding domain. S2 acts as an intermediary in viral fusion in host cell transmission [62].

The N protein is bound to RNA and is involved in processes related to the viral genome, viral replication cycle, viral infection, and the host cellular response [63]. The M protein is the most structured protein and plays a major role in determining the virus envelope shape. Binding with this protein is essential in nucleocapsid or N protein stabilization and promotes completion of viral assembly through stabilization of the N protein–RNA complex inside the internal virion [64]. The function of E protein, which is the smallest protein of the virus, is to participate in the production and maturation of the virus [65].

SARS-CoV-2 is transmitted from human to human via the respiratory route in the form of droplets or direct contact [66]. The results of research, where viral RNA was detected in infected patients’ stool samples, also indicate that attention should be paid to potential fecal–oral transmission. It has been proposed that SARS-CoV-2 may remain longer in the gastrointestinal tract than in the respiratory tract [67,68]. Studies have shown that infected patients were able to excrete viral RNA in feces for a few weeks after the onset of symptoms. Nevertheless, respiratory transmission is the primary route for SARS-CoV-2 [69]. Adsorption of the virus to dust or particulate matter allow the virus to be transported over long distances, especially if the particles carry moisture. In a study conducted in Italy, a high SARS-CoV-2 infection rate was associated with urban air pollution above the limits set for PM_10_ [70]. Another study has suggested that the airborne transmission route of the virus is highly virulent and dominant, so face covering is crucial in reducing the spread of COVID-19 [71].

It is believed that SARS-CoV-2 is zoonotic; however, its origin remains unknown. Pangolins were suggested as a possible virus intermediate host, focusing our attention on the role of animals as reservoirs and vectors of SARS-CoV-2. In order to exclude this hypothesis as a possible transmission routes among wild animals, farm companion animals, and humans, further investigation is needed. Experimental infections have shown a susceptibility to SARS-CoV-2 in mammals from various orders, including rodents (hamsters) [72,73], bats (fruit bats) [74], carnivores (cats, dogs, and ferrets) [74,75,76,77,78], scandent (tree shrews) [79], and non-human primates (African green monkeys, cynomolgus macaques, and common marmosets) [80,81]. Natural infections with SARS-CoV-2 have also been reported in human companion animals and the animals living near humans, such as dogs and cats [82,83,84,85]. Interestingly, a serological study carried out in Wuhan after the outbreak of COVID-19 detected neutralizing antibodies of SARS-CoV-2 in cat serum samples, which implies SARS-CoV-2 infection of the cat population in Wuhan [86]. Some farm animals, such as ferrets and minks, are also susceptible to viral transmission [87,88], and SARS-CoV-2 infections have been reported on mink farms in the Netherlands, Denmark, several other European countries, and the USA [89]. An analyses of SARS-CoV-2 infections in humans living or working on Dutch mink farms indicated cases of human to animal transmission and also a reverse transmission from animals to human with strains with an animal sequence signature [90]. These new strains accumulated changes in the amino acid sequence in the spike surface glycoprotein, which may potentially affect vaccine efficacy [91]. As a result, the Ministry of Environment and Food of Denmark decided to cull all farm minks in the country [92]. Some other countries also considered similar decisions. There is, however, no evidence that other farm animals, such as chickens, ducks, turkeys, quail, geese, or pigs are susceptible to SARS-CoV-2 [74,75,93]. There is also no evidence that arthropods, including hematophagous insects, pose a risk for transmission of SARS-CoV-2 to humans or animals. The studies of mosquitoes (*Aedes aegypti*, *Ae. albopictus*, *Culex tarsalis*, and *C. quinquefasciatus*), biting midges (*Culicoides sonorensis*), known as biological vectors for numerous arboviruses, and also their cell lines, did not support SARS-CoV-2 replication [94,95]. There are also no reports of mechanical transmission of the virus to humans by arthropods living near humans, such as flies and cockroaches. Still the presence of SARS-CoV-2 in the stool of infected humans, as well as some domestic and farm animals, suggests that this route of transmission should be monitored.

## 5. SARS-CoV-2—Mechanism of Action, Clinical Manifestations

**Figure 2:**
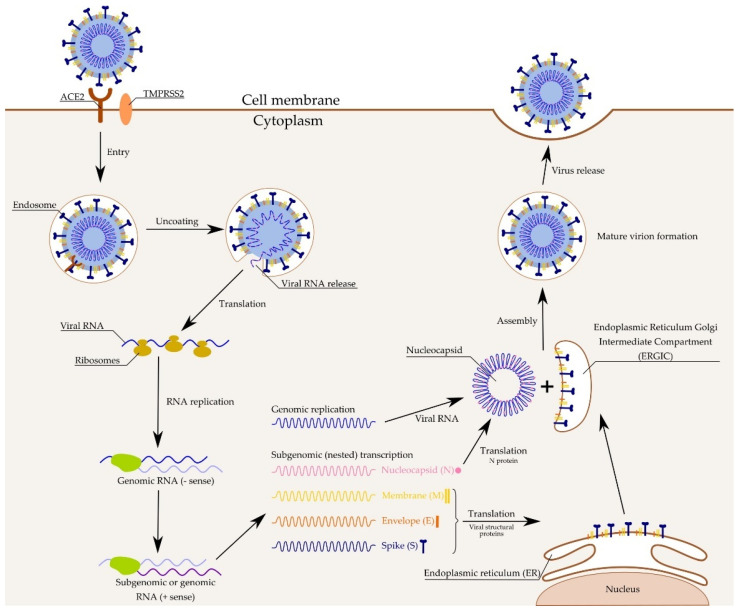
SARS-CoV-2 mechanism of action.

Angiotensin converting enzyme 2 (ACE2) is the main host receptor of SARS-CoV-2 and plays an important role in cellular entry. It has been reported that ACE2 is found in type II lung alveolar cells (AT2), the upper esophagus and stratified epithelial cells, absorptive enterocytes from the colon and ileum, kidney proximal tubule cells, bladder urothelial cells, and myocardial cells [96]. The process of virus entering the cell begins when the S protein of the virus binds to ACE2 in the host cells (Figure 2). Next, the viral membrane is fused with the host cell. After that event, the type II transmembrane serine protease (TMPRSS2), which is present on the surface of the host cell, removes the ACE2 and activates the receptor, attaching like the S proteins. Activation of the S proteins leads to conformational changes and allows the virus to enter the cells through the endosomal pathway. TMPRSS2 and ACE2 are the main determinants of virus entry [97,98,99].

Then viral RNA is released into the cytoplasm and two open reading frames, ORF1a and ORF1b, are translated. The resulting polyproteins pp1a and pp1ab are processed into the individual nonstructural proteins (NSPs) that form the viral replication and transcription complex (RTC) [100]. During replication, RTC drives the production of full length (−) RNA copies of the genome and used as templates for the full-length (+) RNA genomes. During transcription, sub-genomic RNAs (sgRNAs) are produced in a manner of discontinuous transcription [101]. The S, E, and M proteins enter the endoplasmic reticulum (ER) and the N protein are linked with the positive-stranded genomic RNA and form a nucleoprotein complex. The nucleoprotein complex and structural proteins are assembled with a viral envelope at the endoplasmic reticulum—Golgi compartment (ERGIC). Then, the new viral particles are released from the infected host cell [102]. 

The clinical manifestation of COVID-19 involves a broad range of symptoms and patients have symptoms similar to other common flu-like illnesses. Larsen et al. have developed the most probable pathway of COVID-19 symptoms. According to their study, the first is a fever, then cough, and next either sore throat, myalgia, or headache, followed by nausea/vomiting and diarrhea. The authors performed a comparative analysis of the progression of these COVID-19 symptoms with other respiratory diseases like MERS, SARS, and influenza. According to them, COVID-19 differs from MERS and SARS in gastrointestinal symptoms. Influenza starts with a cough, while COVID-19, like other coronavirus-related illnesses, starts with a fever [103]. In severe cases, acute lung injury or a fatal form of acute respiratory distress syndrome (ARDS) is observed [1]. A case report of 99 COVID-19 patients from Wuhan hospital has revealed an increase in the total number of CRP, interluekin-6 (IL-6), and neutrophils—86%, 52%, and 38%, respectively—and a 35% decrease of the total number of lymphocytes [104]. Zhou and colleagues have observed elevated levels of many proinflammatory cytokines in COVID-19 patients, suggesting the pathogenic role of hypercytokinemia. In ARDS patients, the alveolar spaces were occupied by the infiltrating monocytes and neutrophils, indicating the pathogenic role of these cells. Thus, a dominant chemokine, hyperactive cytokine response in COVID-19 cases has also been revealed [105]. In another study, scientists have identified a unique signature of immune dysregulation in patients with SARS-CoV-2. All SARS-CoV-2 pneumonia patients with developed severe respiratory failure exhibit hyperinflammatory responses with features of immune dysregulation or macrophage activation syndrome. Both are characterized by proinflammatory cytokines. The immune dysregulation is driven by IL-6 and macrophage activation syndrome caused by interleukin-1β (IL-1β). Two features of this immune dysregulation are the overproduction of proinflammatory cytokines by monocytes and lymphocyte dysregulation, which is characterized by CD4 lymphopenia and then B cell lymphopenia. Probably, as a result of rapid virus reproduction, the number of absolute natural killer (NK) cells decreases [106]. Lucas et al. have analyzed the immune responses of patients with moderate to severe COVID-19 cases. Immune profiling showed an overall increase in innate cell lineages with reducing the number of T cells. An early promotion of cytokines was linked with a worse disease course. An early rise in cytokines resulted in a progressive reduction in type 1 (antiviral) and type 3 (antifungal) responses in patients with moderate COVID-19. In patients with severe COVID-19, the elevated responses were sustained throughout the disease course. What is more, severe disease was accompanied by an increase in many type 2 effectors like interleukin-5 (IL-5), interleukin-13 (IL-13), immunoglobulin E (IgE), and eosinophils [107].

## 6. Stability of SARS-CoV-2 on Different Surfaces

There have been many studies on the persistence of SARS-CoV-2 on different types of surfaces; these data are summarized in Table 1. Doremalen et al. have demonstrated that the virus was more stable on plastic and stainless-steel surfaces than on copper and cardboard. Viable viruses were detectable up to 72 h after application to plastic and stainless steel, while on copper surfaces no viable viruses were measured after 4 h, nor on cardboard after 24 h [108]. According to another study, dried SARS-CoV-2 on glass surfaces remained viable for 3–4 days at room temperature (22–25 °C) and for 14 days at 4 °C, but quickly loses its viability within 1 day at higher temperatures (37 °C) [109]. 

In a different study that has been conducted at room temperature (22 °C), with a relative humidity of 65%, the virus was not detected on documents and tissue paper after 3 h, nor in treated wood and cloth after 48 h. Interestingly, detectable levels of the infectious virus may still be present on the outer layer of a surgical mask on day 7 [110].

## 7. SARS-CoV-2 Protection Strategy

**Figure 3:**
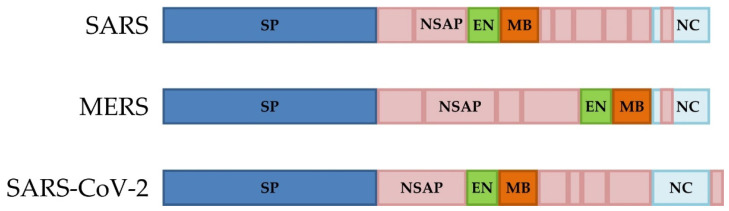
The simplified diagram of SARS, MERS, and SARS CoV-2 genomic organization. SP—spike; NSAP—non-structural and accessory proteins; EN—envelope; MB—membrane; NC—nucleocapsid.

Considerable environmental contamination by COVID-19 patients through respiratory droplets and fecal excretion indicates that the environment is a potential means of transmission; thus, strict adherence to environmental and hand hygiene is needed [111,112]. Until an effective drug is developed or the vaccines against SARS-CoV-2 widely used, all measures to ensure public health must remain consistent in preventing the virus spread through droplets via close contact and contaminated surfaces [113]. Many vaccines were developed against SARS and MERS; however, not a single one was approved for use in humans. Although, knowledge concerning the differences in genomic organization among SARS, MERS, and SARS CoV-2 (Figure 3), and previous experience in vaccine development, allowed to develop the successful vaccine against SARS CoV-2 infection.

As with several other emerging or recurring diseases that are transmitted by aerosols or droplets, it is important to adopt good hygiene practices, like washing hands with soap and water for at least twenty seconds and then using 70% alcohol gel. Furthermore, it is effective to practice social distancing as a preventive measure, avoiding both large crowds and contact with people who may be infected [114,115]. Data on COVID-19, SARS, and MERS provided evidence that the policy of physical distance of at least 1 m may not be sufficient—2 m may be more effective. Moreover, the data also suggest that wearing face masks is important because it protects people (both the general public and healthcare professionals) from infection, and eye protection can bring additional benefits. One of the concerns behind the mass use of face masks is the lack of masks for hospital personnel, who are involved in the direct care of COVID-19 patients and are more exposed [116,117]. At the beginning of the pandemic, the WHO confirmed that the current global inventory of personal protective equipment (PPE), such as medical masks, respirators, gowns, and goggles is insufficient, especially in the case of medical masks and respirators [118]. N95 respirators provide better filtration of airborne particles than medical masks when used correctly and continuously [119]. The CDC has provided a summary of the implementation of Filtering Facepiece Respirator (FFR) reuse during the shortages of N95 respirators. It was explicitly stated that the use of decontaminated FFR should be a part of crisis capability strategy. Ultraviolet germicidal radiation, steam hydrogen peroxide, and humid heat were recommended as potential FFR decontamination methods. It has been suggested that 10–20% of SARS-CoV-2 infections occur among healthcare professionals [120]. Based on experience with other respiratory viruses, correct and consistent use of PPE is crucial in reducing nosocomial transmission [121]. In a study conducted by Liu et al., the authors have examined whether appropriate PPE could protect frontline healthcare workers who are exposed to SARS-CoV-2 infection. Healthcare professionals were equipped with standardized PPE, including protective suits, aprons, masks, goggles, gloves, and face shields. They were also trained in the correct PPE usage and in reducing exposure to infections when caring for COVID-19 patients. The hand washing procedure recommended by the World Health Organization was also strictly followed. Despite the high exposure, no evidence of infection was found in any of the 420 participants. These results indicate that compliance with standard recommendations and appropriate PPE effectively protected hospital personnel from SARS-CoV-2 infection in clinical conditions with a high risk of exposure [122]. An interesting and promising strategy was developed by Campos and colleagues [123]. Wearing PPE for hours while caring for SARS-CoV-2 patients may accumulate viruses on their external surfaces and, without decontamination, may pose a risk to healthcare professionals. Self-decontaminating PPE is a notable solution as it can reduce the risk of contamination from PPE. They used Duritex^TM^ (Ghost, Buda, TX, USA), a natural biopolymer and disinfectant with a complex chemical composition, which they bonded to the fabric in a thermal process. Results demonstrated that Duritex^TM^ can provide the self-disinfecting process and is effective against the virus. This technology also has the potential to be used in different PPE, such as masks or shoe covers, or for self-disinfecting clothing and masks applicable for the general population [123]. A summary of the relationship between the SARS-CoV-2, SARS, and MERS protection strategies is presented below in Table 2.

## 8. SARS-CoV-2 Decontamination Issues

Apart from PPE, an important aspect of actions against COVID-19 is the use of disinfectants. SARS-CoV-2 is not highly resistant to chemical disinfectants, and can be inactivated by physical agents like heat and ultraviolet (UV) radiation. Although, improper use of disinfectants can cause side effects for medical personnel, and improper use with poor ventilation can result in fire, explosion, gas poisoning, or equipment corrosion [124]. Ethanol and isopropanol are the main alcohols that are used as disinfectants for a wide range of viruses, bacteria, and fungi; however, it is important to know that the biocidal activity of both alcohols depends on their concentration and water affinity. Alcohol is believed to damage the cell membrane and denature viral proteins, and also damages the RNA. The strong capability of these alcohols to form hydrogen bonds and their amphoteric nature allow them to disrupt the tertiary structure of proteins by breaking the intramolecular hydrogen bonds in the structure [113,125]. 0.5% hydrogen peroxide can inactivate some coronaviruses, including SARS-CoV-2. Nevertheless, thermal decomposition properties when exposed to heat surfaces is observed [124]. The active chemical in common bleach is sodium hypochlorite, normally present in a concentration range of 3–6%. At low pH (4–7), the hypochlorite anion undergoes protonation and exists in balance with hypochlorous acid, which will be the dominant species. Acid is an active biocide because of its strong oxidizing properties, which damages membrane lipids and nucleic acids. Compounds such as formaldehyde and glutaraldehyde are considered as high-level disinfectants for medical equipment. The use of formaldehyde is limited, because it is listed by Occupational Safety and Health Administration (OSHA) as a potential carcinogen. These aldehydes disinfect viruses and bacteria by alkylating their nucleic acids and proteins and are active against the SARS coronavirus in the concentration range of 0.5–3% within 2 min of exposure [125]. Kampf et al. in their study have shown that ethanol (at the concentration range 78–95%), isopropanol (at the concentration range 70–100%), a combination of 45% isopropanol with 30% n-propanol, povidone iodine (at the concentration range 0.23–7.5%), glutaldehyde (at the concentration range 0.5–2.5%), and formaldehyde (at the concentration range 0.7–1%) successfully inactivated coronavirus infectivity. The effectiveness of sodium hypochlorite required a concentration of at least 0.21%, while hydrogen peroxide was efficient at a concentration of 0.5% and an exposure time of 1 min. Similar effects are expected when using these disinfectants against SARS-CoV-2 [126]. Ultraviolet radiation, which is an electromagnetic wavelength from 10 to 400 nm, is another well-known technique to inactivate microorganisms and viruses. This method has some advantages over thermal sterilization or chemical disinfectants. The whole process can be performed automatically and used to disinfect liquids, surfaces, air, and rooms, and it is energy efficient. According to an analysis of published studies, UVC radiation has been very effective against all coronaviruses. The results include studies of various coronaviruses, including SARS-CoV and MERS-CoV, but not SARS-CoV-2. However, it can be assumed that they also suitable for SARS-CoV-2 and also to all future mutations. It is believed that RNA mutations can have a strong impact on the pathogenicity of the virus, but do not cause major structural differences, especially in the UV RNA absorption properties, which are the main cause of the antiviral activity of UV radiation [127,128].

## 9. Conclusions and Future Perspectives

The world has been struggling with a pandemic for almost a year. In addition to its health effects and health care system impact, the pandemic is affecting the global economy, society, agriculture, tourism, and education. This multidirectional impact of the SARS-CoV-2 pandemic justifies international, collective action and global investment in the development and distribution of preventive measures, such as drugs and vaccines. The recent authorization of two COVID-19 vaccines gives hope to control the spread of the virus. However, approx. 70% of the human population would need to be vaccinated to acquire resistance to SARS-CoV-2 in order to evict the virus. It will take time and will be strongly dependent on vaccine availability, especially in poor countries. Nevertheless, we believe that our common efforts will end the pandemic and the world will learn lessons, especially in terms of public health perspectives, which should be considered globally, especially in case of future pandemics or epidemics.

## Figures and Tables

**Table 1:** Stability of SARS-CoV-2 on various surfaces.

Surface	Persistence	Temperature (°C)	Reference
Paper	3 h	22	[110]
Copper	4 h	21–23	[109]
Cardboard	24 h	21–23	
Treated wood	48 h	22	[110]
Cloth			
Plastic	72 h	21–23	[108]
Stainless steel			
Glass	24 h	37	[109]
	3–4 d	22–25	
	4 d	4	
The outer layer of a surgical mask	7 d	22	[110]

**Table 2:** Summary of the SARS, MERS, and SARS-CoV-2 protection strategies.

Protection Strategy	SARS	MERS	SARS-CoV-2
Policy of minimizing the chances of exposure	YES	YES	YES
Contact and Airborne Precautions	YES	YES	YES
Monitoring the illness	YES/NO	YES/NO	YES
Cases reporting system	YES/NO	YES/NO	YES
Treatment	Symptomatic	Symptomatic	Symptomatic
Vaccine availability	NO	NO	YES
